# Web-based stress management for newly diagnosed cancer patients (STREAM-1): a randomized, wait-list controlled intervention study

**DOI:** 10.1186/s12885-016-2866-0

**Published:** 2016-11-03

**Authors:** Astrid Grossert, Corinne Urech, Judith Alder, Jens Gaab, Thomas Berger, Viviane Hess

**Affiliations:** 1 Medical Oncology, University Hospital Basel, Petersgraben 4, CH 4031 Basel, Switzerland; 2 Obstetrics and Gynecology, University Hospital, Spitalstrasse 21, 4031 Basel, Switzerland; 3 Clinical Psychology and Psychotherapy, University of Basel, Missionsstrasse 62, CH 4055 Basel, Switzerland; 4 Clinical Psychology and Psychotherapy, University of Bern, Fabrikstrasse 8, CH 3012 Bern, Switzerland

**Keywords:** Stress, Depression, Anxiety, Online, Web-based, Cancer, Stress-management intervention, Minimal-contact

## Abstract

**Background:**

Being diagnosed with cancer causes major psychological distress, yet the majority of newly diagnosed cancer patients lack psychological support. Internet interventions overcome many barriers for seeking face-to-face support and allow for independence in time and place. We assess efficacy and feasibility of the first web-based stress management intervention (STREAM: STREss-Aktiv-Mindern) for newly diagnosed, German-speaking cancer patients.

**Methods/design:**

In a prospective, wait-list controlled trial 120 newly diagnosed cancer patients will be included within 12 weeks of starting anti-cancer treatment and randomized between an immediate (intervention group) or delayed (control group) 8-week, web-based intervention. The intervention consists of eight modules with weekly written feedback by a psychologist (“minimal-contact”) based on well-established stress management manuals including downloadable audio-files and exercises. The aim of this study is to evaluate efficacy in terms of improvement in quality of life (FACT-F), as well as decrease in anxiety and depression (HADS), as compared to patients in the wait-list control group. A sample size of 120 patients allows demonstrating a clinically relevant difference of nine points in the FACT score after the intervention (T2) with a two-sided alpha of 0.05 and 80 % power. As this is the first online stress management intervention for German-speaking cancer patients, more descriptive outcomes are equally important to further refine the group of patients with the largest potential for benefit who then will be targeted more specifically in future trials. These descriptive endpoints include: patients’ characteristics (type of cancer, type of treatment, socio-demographic factors), dropout rate and dropout reasons, adherence and satisfaction with the program.

**Discussion:**

New technologies open new opportunities: minimal-contact psychological interventions are becoming standard of care in several psychological disorders, where their efficacy is often comparable to face-to-face interventions. With our study we open this field to the population of newly diagnosed cancer patients. We will not only assess clinical efficacy but also further refine the target population who has the most potential to benefit. An internet-based minimal-contact stress management program might be an attractive, time- and cost-effective way to effectively deliver psychological support to newly diagnosed cancer patients and an opportunity to include those who currently are not reached by conventional support.

**Trial registration:**

ClinicalTrials.gov NCT02289014.

## Background

### Impact of cancer on mental health

Every second cancer patient suffers from clinically relevant psychosocial distress [1]. Psychosocial distress encompasses emotional lability, rearranging of roles and responsibilities, changing of future plans, fear of recurrence, depression and anxiety and is associated with decreased quality of life [2–4]. In addition, high levels of distress lead to reduced compliance with treatment and more side effects [5, 6]. Conversely, side effects of cancer treatment like fatigue, nausea and pain may trigger distress and, therefore, impact psychological adjustment.

### Efficacy of psycho-oncological interventions and Utilization of psycho-oncological support

Cognitive behavioural techniques, including relaxation techniques [7] and mindfulness based stress reduction [8], significantly reduce distress, depression and fatigue and increase quality of life in cancer patients, albeit effect sizes in randomized controlled trials are small to medium [9]. Moreover, psycho-oncological interventions may reduce side effects of cancer treatment [7, 9–13]. Yet many patients do not seek or have access to psycho-oncological support, even when high levels of distress are experienced [14]. This seems especially true for male patients [15, 16] and patients with cancer other than female breast cancer [15].

### Web-based interventions in psycho-oncology

The internet has the potential to reach patients and to overcome barriers towards using psycho-oncologic support (e.g. stigma and privacy concerns, geographical distance form providers, time constraints to adhere to additional appointments during office hours) [17]. The vast majority of cancer patients already uses the internet as a source of information [18]. Furthermore, from the providers’ perspective, internet interventions are time- and cost-effective, and thus are of special interest for the health care system. Scientific interest in internet interventions for non-cancer patients has grown rapidly over the last decade. Efficacy of this novel treatment format has been demonstrated for a variety of mental disorders in a substantial number of randomized controlled trials (RCTs). Reviews and meta-analyses show moderate to large effects, post-treatment [19–22]. Studies directly comparing internet interventions with face-to-face therapy report similar outcomes across various mental disorders (e.g. anxiety disorders, depression) and health concerns associated with bodily symptoms (e.g. tinnitus, sexual dysfunction) [23]. There are also a few long-term follow-up studies showing lasting effects over as much as five years post-treatment [24]. Data on web-based interventions for cancer patients are scarce [25, 26]. There is no cancer-specific stress management program for cancer patients in German. Also, little is known on the characteristics (including age, sex, education, type of cancer) of patients who participate and benefit from a web-based intervention.

### Objective and research questions

In a prospective randomized wait-list controlled trial we assess the efficacy of a minimal-contact online intervention in newly diagnosed cancer patients. More specifically, we assess whether patients who undergo the online intervention report a better quality of life (FACIT-F), are less anxious and depressive (HADS-D), less stressed (DT), and cope better with their disease (FAH II) as compared to patients in the wait-list control group. This is the first online stress management intervention in German for cancer patients (STREAM: **STRE**ss **A**ktiv **M**indern). Moreover, since it is novel to recruit patients via the internet rather than face-to-face, we set out to determine patients characteristics for participation and benefit. These descriptive outcomes include: patients’ characteristics (type of cancer, type of treatment, sociodemographic factors), patients’ adherence as well as satisfaction with the program.

## Methods/design

**Fig. 1:**
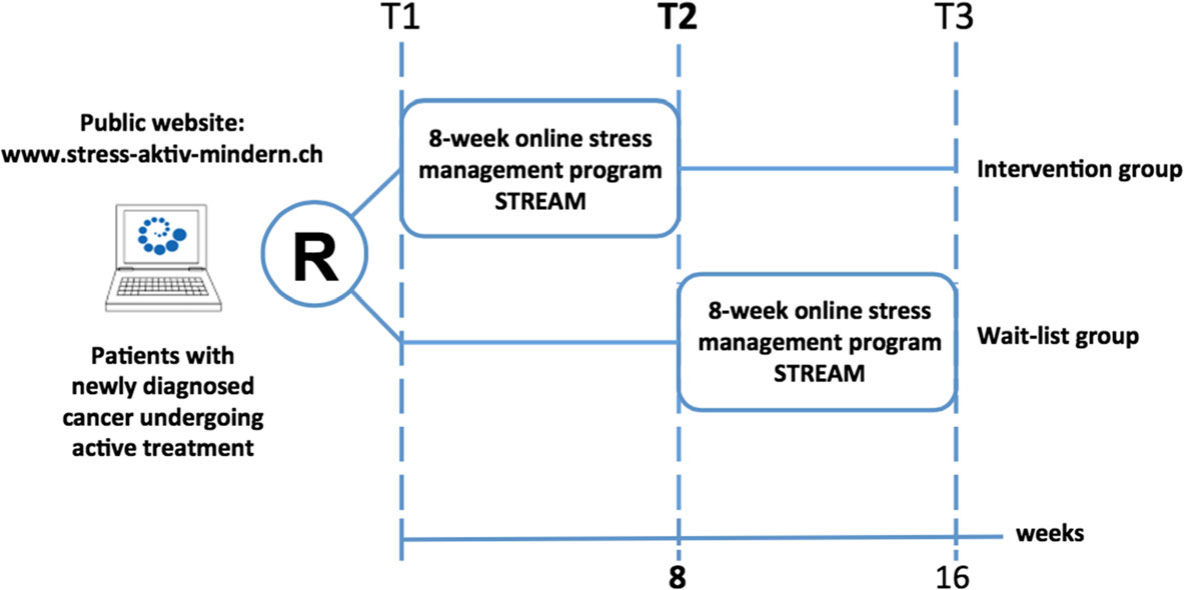
Study design

In a prospective randomized controlled intervention study (Fig. 1) patients are randomized 1:1 (mixed randomization scheme using unequal block randomization) between the intervention group and the wait-list control group. Patients are stratified according to baseline stress level (distress thermometer ≥ *vs* < 5 [27]). A total of 120 newly diagnosed adult (>18 years) cancer patients who started first-line treatment (either systemic treatment - including chemotherapy, hormonal treatment or targeted therapy - or radiotherapy) no longer than 12 weeks earlier are included after giving informed consent. Patients who undergo treatment for a first relapse of a tumor previously treated with curative intent are also eligible. Patients are required to read and write in German, have internet-access as well as basic computer skills.

Patients are recruited via the public website of STREAM [28]. We adopt an *active* recruitment strategy in the German speaking countries Switzerland, Germany, and Austria via the following channels: a) Links to the public website of STREAM on health related websites, such as Cancer Leagues, Cancer Hospitals, Patient advocate websites b) distribution of flyers in hospitals and during cancer conferences, c) active communication to the media d) advertisement via Google Ad and Facebook Ad. The Northwest and Central Swiss Ethics Committee has approved the study (EKNZ 339/13).

### Intervention

We developed the web-based intervention STREAM based on well-described and established stress management interventions manuals [29–33], which we adapted to the web-context. The program aims at improving intra- and interpersonal coping strategies, reducing perceived stress and anxiety as well as enhancing quality of life. We assessed the usability of the program in the target population of cancer patients in a previous study (Grossert A, et al. Usability evaluation of the web-based stress management program STREAM for newly diagnosed cancer patients. submitted. 2016).

STREAM consists of eight modules (Table 1) which can be completed in 60 to 90 min each. Participants are asked to work through one module per week, i.e. the intervention takes 8 weeks in total. Every module starts with a short mindfulness breathing exercise and includes (a) text-based psycho-education, (b) reflection on the current individual emotional status and (c) acquisition of strategies including several exercises. Within each module exercises on relaxation techniques and guided-imagery exercises are available as audio files and can be downloaded to various devices (e.g. PC/Mac, tablets, smart phones, MP3 Players). Patients are encouraged to use the audio files daily. Participants receive weekly feedback and individual support by a psychologist from the study team via secure integrated e-mail (=”minimal-contact”). Patients can use the secure chat function to contact the psychologist.

**Table 1:** Content of the web-based stress management program STREAM

Module		Psychoeducation	Reflection on status	Strategies
1.	Introduction: What is stress?	Nature of stress in general and in specific cancer-related situations	My individual stressors	Diary^b^
			Stress protocol	Body scan^a^
				Stress protocol ^b^
2.	Body stress reduction	Bodily sensations during stress and side effects of anticancer treatment	Relaxation protocol	Progressive muscle relaxation ^a^
				Beach promenade^a^
				Relaxation protocol^b^
3.	Cognitive stress reduction	Thoughts and their interaction with emotions and bodily sensations	My negative thought patterns	Negative thought cycle^a^
				Relationship of body position and thoughts^b^
				Thinking styles and reflection^b^
				Thoughts on clouds^a^
4.	Emotional stress reduction	Feelings and cancer-related emotions as anxiety and worries were explained	My feelings and worries	Mountain meditation^a^
				Emotional emergency kit^a^
5.	Mindfulness and acceptance of thoughts and emotions	Meaning of mindfulness and acceptance and their implementation in daily life in contrast to the active strategies learned in modules 1–4	My definition and experiences with acceptance	Acceptance story ^a,b^
				Body scan^a,b^
				Integration of mindfulness^b^
				Winter walk^a^
				Spring awakening^a^
6.	Activation of resources: quality of life and pleasure	Introduction of models of the balance between burden and resources	My individual resources	Health-cycle ^b^
				Planning activities^b^
				Week planner ^b^
				Friendly feelings with our own body^a^
				Enjoyment training^b^
7.	Activation of resources: social network and communication skills	Social network and the role of a supportive environment	My individual social network and current needs	Communication skills^b^
				Beach promenade^a^
				Winter promenade^a^
				Spring awakening^a^
8.	Summary	Concluding an overview and documentation of the last 7 weeks	My experiences with the program	Four seasons^a^

^a^Audio file: story, relaxation or guided imaginary exercise, ^b^Instructions and worksheets

### Assessments

Assessments are summarized in Table 2 and are conducted via an open source survey application [34] at baseline (T1), after the 8-week intervention or wait (control group), respectively (T2), as well as after an additional 8 weeks (T3, follow-up for the intervention group or after the delayed intervention for the wait-list group, respectively (Fig. 1)).

**Table 2:** Schedule of assessment

Instrument	T1		T2	T3
	Baseline	Weekly during intervention	Post intervention	Follow up
Socio-demographics	x			
Medical history	x		x	x
Distress Thermometer (DT)	x	x	x	x
Functional Assessment of Chronic Illness Therapy-Fatigue (FACT-F)	x		x	x
Hospital anxiety and depression scale (HADS)	x		x	x
Suicidal tendency (Suicid Item Beck Depression Inventory BDI)	x		x	x
Acceptance and Action Questionnaire (AAQ)	x		x	x
Working Alliance Inventory (WAI-SR)		x		
System Usability Scale (SUS)		x^a^		
Client Satisfaction Questionnaire (CSQ-8)			x^b^	x^b^

^a^ Assessed twice (after the first and last module); ^b^ Assessed post-intervention: for intervention group to T2, for control group to T3

**Socio-demographic** information is self-reported and includes age, gender, marital status and partnership, children, education, monthly household income and employment status.

**Medical history** includes information regarding tumor diagnosis, time since diagnosis, past and current cancer treatments (curative or palliative setting), past and current psychosocial support and psychopharmacological medication and is obtained from both patients and their treating physicians.

### Efficacy outcomes

The main efficacy outcome is quality of life (FACT) including assessment of fatigue (FACT-F) [35] at T2. We use the validated German Version **functional assessment of chronic illness therapy-fatigue (FACIT-F)** which is freely available from the website www.facit.org.

To assess anxiety/depression and psychological distress the ** hospital anxiety and depression scale (HADS)** [36] and the **distress thermometer (DT)** in the German version [27] are used, respectively. Patients are stratified based on their level of baseline distress. A score of five or higher at the DT visual analogue scale is a cut-off score for a clinically significant level of distress [27]. To longitudinally describe psychological coping with cancer we use ‘the acceptance and action questionnaire (AAQ)’ in its German version ‘***Fragebogen zur Akzeptanz und Handeln*** **(FAH II)’** [37], which we adapted specifically for cancer patients with three additional items concerning their coping with the disease.

The online support program STREAM is not designed to support suicidal patients in acute crises. For safety reasons, **suicidal tendency** is assessed by the single suicide item out of the Beck Depression Inventory (BDI) [38]. Patients with a score higher than one are contacted by telephone to reassess suicidal ideation and, if needed, patients are instructed to call for local psychiatric support.

### Evaluation of the intervention

To evaluate the therapeutic alliance between patient and therapist ﻿the ** short version of the working alliance inventory (WAI-SR)** with subscales for bond, tasks and goals is used in its German version [39] weekly after each module. Usability and user satisfaction is assessed with the **system usability scale (SUS)** [40] and the Client Satisfaction Questionnaire (CSQ-8; in its German version: *Fragebogen zur Messung der Patientenzufriedenheit* ZUF-8; [41]. Satisfaction with the online therapeutic contact will be assessed with predefined questions described by Knaevelsrud and Maerker [42]. In addition, after each module, patients’ satisfaction with the module is assessed with an open question. Data on adherence (frequency and duration of logins, website activity, using/downloading different exercises, and the number of modules completed) are collected via the backend functions of the online program STREAM.

### Statistical analyses and sample size calculation

Based on previous studies [43] including recent data on cancer patients [44], a difference of nine points in the FACT score is both, clinically significant and realistic. In order to demonstrate a 9-point difference between baseline and T2 (after 8 weeks) in the intervention group with a statistical power of 0.80 at a significance level of 0.05 (two-sided), 60 participants are needed in each of the two conditions. We assume normally distributed data in both groups with a standard deviation of ±18 [45]. Data preparation of all continuous dependent variables will include tests for normality, homogeneity of variances, and examination of outliers. If not normally distributed, variables will be subjected to adequate transformation. Intent-to-treat samples will be used to analyze data. The choice of statistical approach depends on the amount of missing data at T2 and T3. If less than 12 % of data are missing, the Last Observation Carried Forward (LOCF) method will be applied to estimate effects. Then, the outcome will be computed with an analysis of covariance (ANCOVA), using the pre-scores as a covariate and the post-scores as the dependent variable. If more than 12 % of data are missing, we will use linear mixed models. This method is recommended for intent-to-treat-analyses with a high amount of dropouts due to its potential to reduce bias caused by missing data. Regression analyses will be used to identify predictors of treatment outcome.

### Discussion

Psychological distress associated with cancer diagnosis and treatment is high. Yet, psycho-oncological support is often lacking –due to barriers on the patients’ side or to insufficient resources on the providers’ side [15]. Online-interventions with regular psychologist-contact (minimal-contact) -already established in several psychological disorders- might reduce this gap. In our prospective, randomized controlled study we assess the first minimal-contact, online stress management program for German-speaking, newly diagnosed cancer patients.

Our study will yield information on the efficacy of the intervention with respect to quality of life and stress/anxiety. In addition, it will show whether cancer patients are ready to use new technologies to further increase the range of treatment options at their disposal, and -even more importantly- whether patients who are in need of support but slip through the net of the current system can be reached. Conversely, the time- (and indirectly the cost-) effectiveness of administering support in a minimal-contact online intervention will be assessed from the providers’ perspective – an outcome with important implications for the health care system.

On a different level, the contribution of various and novel recruitment strategies (flyers, “conventional face-to-face”, internet links, Google Ads, Facebook Ads, You tube) will be described allowing for conclusions for future online study portals.

One of the limitations of the study is the heterogeneity of newly diagnosed cancer patients with respect to tumor type, treatment type and treatment strategies (curative, palliative). However, the distress of a new cancer diagnosis is their common denominator, and a stratification factor. Also, since this is the first online stress management program for newly diagnosed cancer patients, we deliberately aim at reaching a broad population to avoid missing a small but important group of patients with potential benefit from this intervention. The results of this study will allow characterizing the patient population(s) with respect to age, sex, diagnosis and treatment that will then be studied more specifically in follow-up trials. Our trial represents a first step in expanding the much-needed psychological support for newly diagnosed cancer patients towards the promising approaches that come with new technical possibilities which have become integral part of our lives.

### Trial status

Trial start date: 1^th^ July 2014; Currently recruiting (N_current_ = 80 as of March 17, 2016).

## Abbreviations

AAQ: Acceptance and action questionnaire
ANCOVA: Analysis of covariance
BDI: Beck depression inventory
CSQ: Client satisfaction questionnaire
DT: Distress thermometer
FACIT-F: German Version Functional Assessment of Chronic Illness Therapy-Fatigue
FACT (−F): Functional Assessment of Cancer Therapy including Fatigue
FAH II: Fragebogen zur Akzeptanz und Handeln
HADS: Hospital anxiety and depression scale
LOCF: Last observation carried forward
RCT: Randomized controlled trial
STREAM: STREss Aktiv Mindern
SUS: System usability scale
WAI-SR: Short version working alliance inventory
ZUF-8: Fragebogen zur Messung der Patientenzufriedenheit
